# Nutrient Intakes in Early Life and Risk of Obesity

**DOI:** 10.3390/ijerph13060564

**Published:** 2016-06-06

**Authors:** Marie Françoise Rolland-Cachera, Mouna Akrout, Sandrine Péneau

**Affiliations:** Université Paris 13, Equipe de Recherche en Epidémiologie Nutritionnelle, Centre de Recherche en Epidémiologie et Statistiques, Inserm (U1153), Inra (U1125), Cnam, COMUE Sorbonne Paris Cité, Bobigny F-93017, France; mounakrout@yahoo.fr (M.A.); s.peneau@uren.smbh.univ-paris13.fr (S.P.)

**Keywords:** early nutrition, child’s growth, obesity, leptin, metabolic programming, adiposity rebound, epidemiology, secular trends

## Abstract

There is increasing evidence that environmental factors in early life predict later health. The early adiposity rebound recorded in most obese subjects suggests that factors promoting body fat development have operated in the first years of life. Birth weight, growth velocity and body mass index (BMI) trajectories seem to be highly sensitive to the environmental conditions present during pregnancy and in early life (“The first 1000 days”). Particularly, nutritional exposure can have a long-term effect on health in adulthood. The high protein-low fat diet often recorded in young children may have contributed to the rapid rise of childhood obesity prevalence during the last decades. Metabolic programming by early nutrition could explain the development of later obesity and adult diseases.

## 1. Introduction

There is now clear evidence that nutritional and metabolic exposure during critical periods of early human development (“the first 1000 days”) can have a long-term effect on health in adulthood. Early environmental factors can permanently change the structure and function of the body, a phenomenon known as “programming” [1]. Various studies have examined the influence of early nutrition on later obesity risk but there is still no consensus about the role of the different nutrients. A protective effect of breast feeding has been demonstrated in various studies [2]. Several factors may account for this benefit, but the nutrient composition of human milk, which is characterized by a high content of fat and low content of protein, could also play a role. Studies examining associations between nutritional intakes and growth processes provide useful information on the early determinants of childhood obesity. 

## 2. Trends of Childhood Obesity and Nutritional Intakes

Assessment of nutritional status [3] and secular trends of thinness and obesity are essential to monitor health of populations, particularly in children. After a steep increase in childhood obesity, a plateau or even a decline in prevalence rates has been reported in many industrialized countries. While prevalence of childhood obesity was increasing, energy intake was decreasing [4]. This time trend was reported in children and adolescents but also in young children. Decreased energy intake mainly resulted from decreased fat intake. During the period when fat intake was decreasing, the percentage of energy from protein rose, the percentage from fat fell and the percentage from carbohydrates stayed the same. This was observed in many countries such as France, England, Germany or the U.S. [4]. For example, between 1967 and 1993, the period corresponding to the start of the steep increase of childhood obesity, energy intakes in 1.5–2.5 year old English children fell from 1264 to 1045 kcal/day. Such findings contrast with recent trends showing that while obesity was stabilizing, macronutrient pattern appeared to be stable over time [5,6]. 

Decreasing consumption of fat and increasing consumption of proteins was the consequence of replacing whole milk by low fat milk [4]. In 1973 in France, most 2 year-old children consumed whole milk, but 13 years later most milk given to young children was low fat. The main reason for using low fat dairy products could be the belief that they are healthier for the infants [7]. The impact of such choice is very important, as dairy products are the main source of energy in young children. Besides decreasing fat intake, the use of low fat milk increases the contribution of protein in the diet. Whole milk contains 20% of energy from proteins, while semi-skimmed contains 28% and skimmed milk 39% [8]. The composition of low fat milk is very different from the high fat-low protein composition of human milk. The trend of diets with lower energy density and higher protein content may have contributed to the decreased energy intake reported during the last decades. 

The main explanation usually proposed to account for the trend of decreasing energy intake is decreasing energy expenditure. Low physical activity is indeed an important factor, however, this hypothesis seems less convincing in very young children. Other factors may explain the rising trend of obesity.

## 3. Early Nutrition and Later Metabolic Risks

Early nutrition is associated with metabolic risks through its impact on growth processes and hormonal status [4].

### 3.1. Nutrient Impact on Growth Parameters

**Figure 1:**
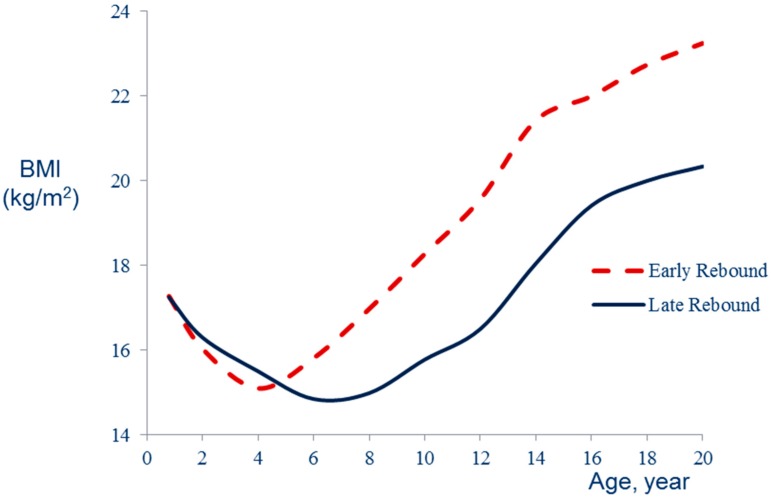
Body mass index (BMI) (kg/m^2^) development according to age (year) at adiposity rebound (after [3]).

**Figure 2:**
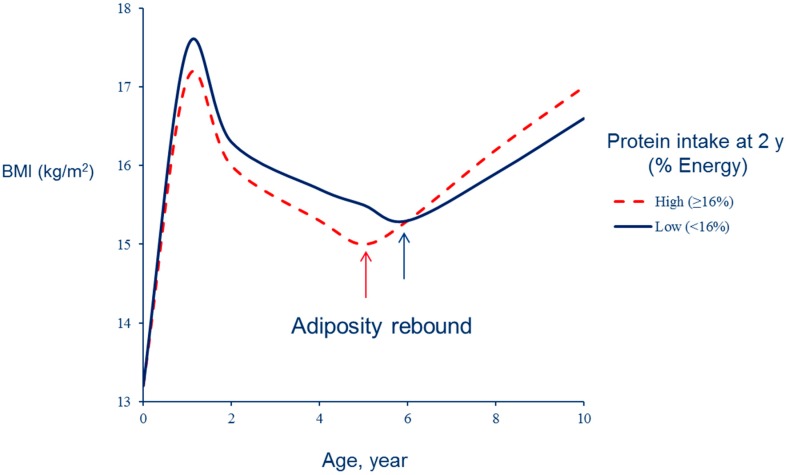
BMI development according to protein intake at 2 years (Etude Longitudinale Alimentation Nutrition et Croissance des Enfants (ELANCE) study) (after [9]).

An association between high protein intake in early life and increased body fat in later development was reported two decades ago in the Etude Longitudinale Alimentation Nutrition et Croissance des Enfants (ELANCE) study [9]. High protein intake was also associated with an earlier adiposity rebound. The adiposity rebound corresponds to the nadir of the BMI curve occurring by 6 years on average but earlier in subjects who will subsequently become overweight [3] (Figure 1). In a first phase, high protein intake is associated with lower body adiposity but, after an earlier adiposity rebound, BMI increases more rapidly (Figure 2). 

Many studies have confirmed the association between early protein intake and later risk of obesity [4,8]. A systematic review of the literature on protein intake from 0 to 18 years of age [10] found convincing evidence that higher protein intake before the age of 2 years was associated with increased growth and higher subsequent BMI, but found limited/inconclusive evidence that protein intake during childhood and adolescence was associated with later risk of becoming overweight, stressing the particular impact of nutrition in early life on the risk of later becoming overweight.

More recently, the ELANCE study investigating the association between early nutrition and adult measurements showed that early low fat intakes were associated with adults being overweight and having high serum leptin [4,11], but there is less evidence for the effects of early fat intake on the growth processes. However, it was shown that infants that were exclusively or predominantly breast fed and consumed higher amounts of fat grew more rapidly in weight and length during the first months, but appeared to falter thereafter [12]. The negative association found between lipid intake in early life and later increase of body fat is in accordance with the conclusion of several reviews of the literature showing no deleterious consequences of high consumption of lipids during the first two years of life [13], or even a protective effect of high-fat dairy consumption [14].

The impact of nutritional conditions on BMI trajectories can be suggested by examining the trends in BMI growth curves. Data from the Fels study have shown that BMI curves of children born during the obesity epidemic were characterized by lower BMI values before the adiposity rebound and by rapid subsequent BMI gain [15]. This trajectory, similar to the patterns of early AR or high protein intake (as shown in Figure 1 and Figure 2), is particularly associated with metabolic diseases such as diabetes [16] or cardiovascular diseases [17]. This secular trend of BMI pattern could be related to the trend of increasing protein and decreasing energy density of the diet of young children [4]. 

### 3.2. Nutrient Impact on Hormonal Status

The association between high protein intake in early life and later obesity may occur through increased growth factors stimulating growth [18] and promoting the proliferation of adipocytes. The association of early low fat intake with being overweight later and having high serum leptin at adult age suggests a programming of leptin resistance [4,11]. 

The adverse effect of reducing fat intake is consistent with the results of studies conducted in different contexts. Low birth weight, which is associated with low leptin concentration [19] or early undernutrition leading to stunting [20], are associated with increased body fat and leptin in the long term [21,22], suggesting leptin resistance. Several mechanisms can account for these associations. Nutrition can act directly on body composition through energy balance [23] or a decrease of fat oxidation, but also indirectly through hormones regulating body weight [24]. A “Low fat programming” was then proposed [11]. In a first phase, a low energy dense diet as a consequence of fat restrictions, may decrease serum leptin concentration, thus programming compensatory metabolic responses promoting later obesity and leptin resistance. These effects can be mediated at least in part through epigenetic processes [1]. The beneficial role of leptin in early life was suggested by the substantial amount of leptin in breast milk [25] and by animal studies showing that the consequences of maternal undernutrition could be reversed by injecting leptin during the suckling period [26]. 

## 4. Breast Feeding and Complementary Feeding

There are many factors which could account for the protective effect of breast feeding. The nutrient composition of breast milk could explain the association between breast feeding and reduced risk of becoming overweight [27]. The later adiposity rebound that was recorded in breast fed children [28], who ingested less protein and more lipids, suggests such a role of early nutrition. However, the protective effect of breast feeding against obesity is still controversial [29]. One of the reasons for the contradictory results may be due to missing confounding factors taken into account. For example, adjustment for the type of complementary foods given to the infant is rarely performed in the analysis examining the association between breast feeding and being overweight later. In a recent study [30], it was shown that inadequate nutrient intake after breast feeding could compromise the beneficial effect of human milk. Low fat diets given after breast feeding can suppress its positive effect, while after adjustment for early nutrition, the beneficial effect of breast feeding clearly appeared. It was observed that mothers who breast fed their child were also more likely to restrict fat intake at weaning, perhaps because they were more aware of the child’s health and used this strategy because they believed it was better for the child’s health [7]. In addition, as the choice of complementary foods introduced in infancy affects food preferences in later life [31], it could be feared that high intakes of fat in infancy could promote excessive fat consumption later on. However, early intakes of fat do not seem to alter later preferences, as fat intake at 10 months was not associated with fat intake at 8 years [32].

## 5. Nutrient Balance in Early Life and Official Recommendations

The positive association recorded between early protein intake and later body fat, and the negative association recorded between early fat intake and later body fat have highlighted the inadequate nutrient balance of the infant diet in industrialized countries. Protein intake represents about 3–4 times the protein needs at this age and fat intake is remarkably low in many countries [4,8,11]. This nutrient imbalance contrasts with the high fat-low protein content of human milk and with official recommendations that fat intake should not be restricted before the age of 3 years [33]. High fat diets are important during early life, when energy needs are very high for growth and for the rapid development of the nervous system.

**Figure 3:**
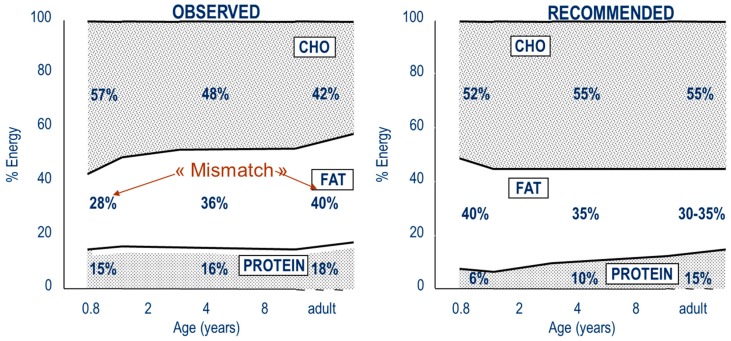
Actual nutrient consumption according to age (ELANCE Study) [11], and recommended intakes.

**Figure 4:**
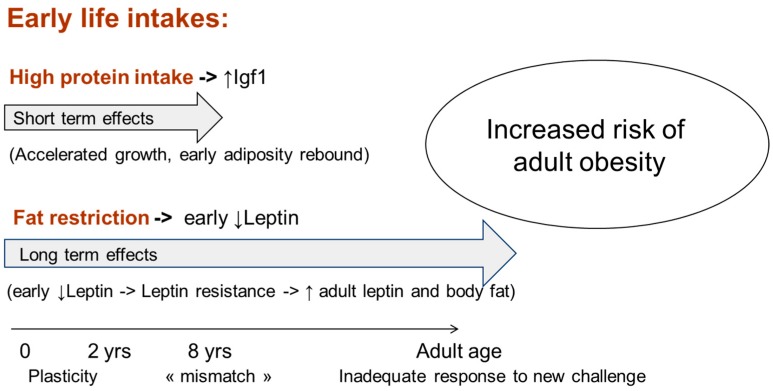
Short and long term effects of imbalance diet in early life.

Paradoxically, fat intake increases with age, while it should be high in infancy and decrease thereafter (Figure 3). In the ELANCE study, fat intake was low in early childhood and subsequently increased with age. Mean energy from fat was 28% at 10 months, 32% at 2 years and 38% from the age of 8 years until adulthood [11]. This “mismatch” [34] between early low and later high fat intake may promote the development of obesity and metabolic diseases. High protein and low fat dietary intake in early life seem to have deleterious consequences, but with a different timing (Figure 4). The effects of high protein intakes on growth appear on the short term (rapid growth and early adiposity rebound, both risk factors for later diseases), likely through an increase of growth factors [18,35], while the consequences of fat restrictions on body composition appear only in the long term (increased body fat at adult age) via the progressive development of leptin resistance, likely due to decreased serum leptin in early life. 

## 6. Conclusions

Evidence suggests that an imbalanced diet in early life can have deleterious consequences on body composition and health. The high protein-low fat diet recorded in young children may have contributed to the obesity epidemic. The consequences of inadequate nutrition at different ages stress the importance of providing nutritional intakes adapted to the child’s metabolic needs at the various stages of growth.
